# Vascular Homeostasis and Angiogenesis Determine Therapeutic Effectiveness in Type 2 Diabetes

**DOI:** 10.1155/2011/971524

**Published:** 2011-05-24

**Authors:** Narisa Futrakul, Prasit Futrakul

**Affiliations:** Renal Microvascular Research Group, Faculty of Medicine, Chulalongkorn University, Bangkok 10330, Thailand

## Abstract

Under common practice, recognition and treatment of type 2 diabetic nephropathy (DN) are usually revealed at a rather late stage (CKD stages 3–5) due to the insensitiveness of available diagnostic markers. Accumulating data obtained from vascular homeostasis in late stage DN demonstrated (1) a defective angiogenesis and impaired NO production which explains the therapeutic resistance to vasodilators and the inability to correct chronic renal ischemia and (2) an abnormally elevated antiangiogenesis and a progressive vascular disease which correlates with the altered renal hemodynamics characterized by a progressive reduction in renal perfusion as the disease severity progressed. In contract, the vascular homeostasis is adequately functional in early stage DN. Thus, vasodilator treatment at early stage DN (CKD stages 1-2) can enhance renal perfusion, correct the renal ischemia, and restore renal function.

## 1. Problems Relevant to Present Therapeutic Failure in Diabetic Nephropathy under Current Practice

**Figure 1:**
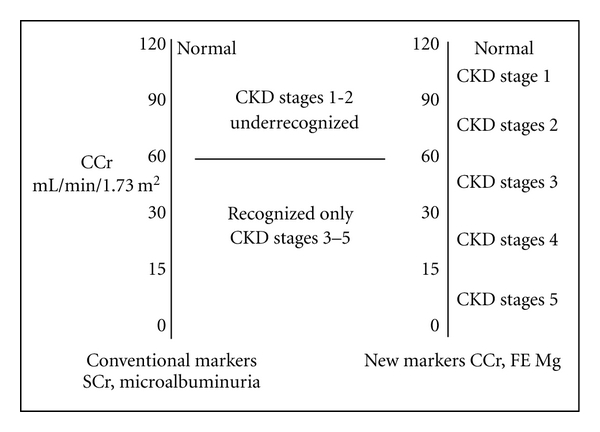
Comparison between conventional and newdiagnostic markers.

Under current practice, determinations of serum creatinine and microalbuminuria are used to screen for diabetic nephropathy (DN), which is reflected by microalbuminuria (30–300 milligrams of urinary albumin/gram creatinine, or greater) and serum creatinine concentration greater than 1 mg/dL. In accordance with the 5 stages of chronic kidney disease (CKD), serum creatinine usually does not change at the early stage (CKD stages 1-2; creatinine clearance 60–119 mL/min/1.73 m^2^), but becomes abnormally elevated only when the creatinine clearance drops to the level of fifty percent (CKD stages 3–5; creatinine clearance <60 mL/min/1.73 m^2^). Similarly, microalbuminuria is usually detected when the creatinine clearance is approaching fifty percent level as depicted in Figure 1 [1, 2]. This implies that under current practice, these diagnostic markers do not recognize diabetic patient at the early stage and we allow these patients to progress towards late stage of CKD without any therapeutic intervention. In contrast to this current practice, new diagnostic markers such as (1) measured creatinine clearance would recognize all stages of CKD and (2) FE Mg, which has previously been demonstrated to correlate directly with the magnitude of tubulointerstitial fibrosis, would recognize early stage of CKD with a mildly impaired renal function [3]. Enhanced excretion of urinary magnesium resulting in abnormal FE Mg is due to the increased detachment of magnesium attached to the ATPase enzymes in the tubular cell following the tubulointerstitial disease. Therefore, these new diagnostic markers assist to differentiate the diabetic patients at the early stage (normoalbuminuria) from the normal population. We have recently demonstrated that diabetic patients in this stage of normoalbuminuria have values of serum creatinine, and urinary microalbumin/creatinine ratio not significantly different from the normal controls. (Table 1) However, these diabetic patients would be easily recognized by (1) measured creatinine clearance which is significantly impaired, (2) FE Mg value doubling the value of normal control, which reflects the presence of tubulointerstitial fibrosis, and (3) evidence of renal ischemia which is reflected by the reduction in peritubular capillary flow.

The insensitiveness of the available diagnostic markers is responsible for the present therapeutic failure in restoring renal perfusion and function. Vasodilators treatment initiated at the late stage of DN is characterized by therapeutic ineffectiveness. In this regard, we have recently demonstrated that such therapeutic failure correlates with the progressive reduction in peritubular capillary flow. This would raise an interesting question as to whether what would be responsible for such therapeutic unresponsiveness to vasodilators in late stage of DN.

## 2. Vascular Homeostasis in Normal (Physiologic) and Diabetes (Pathologic)

Normal vascular homeostasis is the balance between vascular injury and vascular repair. Vascular injury is usually induced by circulating triggers such as altered shear stress [4, 5], oxidative stress [6], cytokine [7], and angiotensin I (AII) [8]. It is reflected by endothelial cell loss from the vascular wall into the circulation, the so-called circulating endothelial cell. This circulating endothelial cell is associated with receptor-bound VEGF as suggested by Hohenstein [9]. We, therefore, have studied circulating endothelial cell-receptor-bound VEGF in normal controls and diabetic patients. With respect to VEGF receptors, there is much debate about their active roles (VEGFR1, VEGFR2) whether which VEGF receptor plays the pathologic role in the progression of renal microvascular disease in diabetes. In answering this question, we have compared the change in renal hemodynamics which is reflected by the reduction in peritubular capillary flow, with the change in VEGF receptors, to explain the renal microvascular disease progression. During the early stage of DN (normoalbuminuria), there is evidence of renal microvascular disease which is reflected by the reduction in peritubular capillary flow. However, the vascular homeostasis in this stage reflected by both VEGFR1 and VEGFR2 is not significantly different from the control, indicating a fairly compensatory status of vascular repair, despite the presence of a mild degree of renal ischemia as depicted in Table 2. During the late stage of DN, there is a progression of renal microvascular disease which is reflected by the further reduction in peritubular capillary flow. This hemodynamic change correlates with the defective VEGFR1 and the abnormally elevated level of VEGFR2. Such changes imply that the predominant role of VEGFR2 determines the progression of renal microvascular disease, and, therefore, VEGFR2 is considered to be antiangiogenic whereas VEGFR1 is angiogenic.

## 3. Vascular Homeostasis under Physiologic Condition

**Figure 2:**
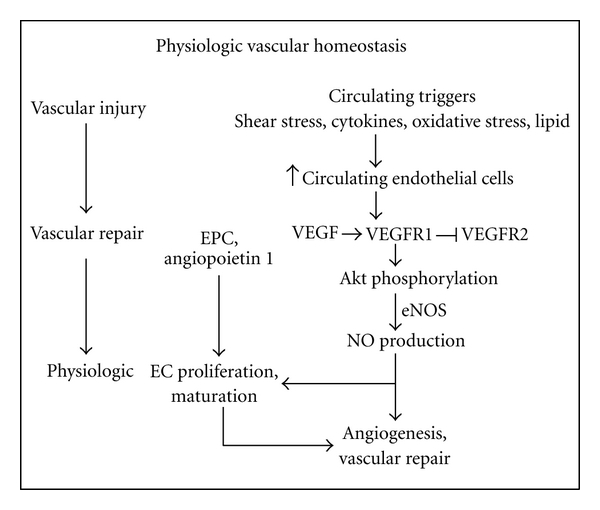
Vascular homeostasis under physiologic condition.

The study of vascular homeostasis in the controls reveals normal values of both angiogenic factors, namely, VEGF, VEGFR1, endothelial progenitor cell, and angiopoietin 1, and antiangiogenic factors, namely, VEGFR2 and angiopoietin 2. In response to vascular injury under physiologic condition, both VEGFR1 and VEGFR2 are essential in coordinating endothelial cell assembly [10]. Vascular repair would recruit (I) VEGF to activate through VEGFR1, whereas activation through VEGFR2 is physiologically suppressed due to the high affinity of VEGFR1. This would physiologically induce Akt phosphorylation, coupling of endothelial nitric oxide synthase (eNOS), and enhance NO production. Previous studies showed that (i) short-term activation of endothelial Akt phosphorylation leads to increased NO production, re-endothelialization, angiogenesis, and vascular protection [11]. (ii) VEGF to activate eNOS via VEGFR1 and that the NO produced then negatively regulates endothelial cell proliferation by VEGFR2 while promoting endothelial cell tubular formation and differentiation into capillary networks [12, 13]. (II) endothelial progenitor cell to the site of vascular injury and angiopoietin 1 in conjunction with the enhanced NO production to corporate in physiologic stimulation of endothelial cell proliferation and maturation. Previous studies demonstrated that exogenous recombinant angiopoietin 1 enhances the growth of interstitial capillaries in mouse metanephric organ culture [14], and the factor enhances transendothelial electrical resistance in monolayer cultures of glomerular endothelial cell [15]. Collectively, all these factors integrate in fine-tuning the angiogenic response to vascular endothelial growth factor, resulting in an adequate vascular repair and normal angiogenesis (Figure 2).

## 4. Pathologic Vascular Homeostasis in Late Stage of DN

**Figure 3:**
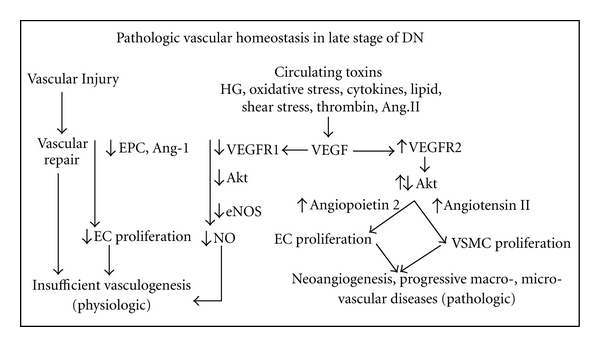
Pathologic vascular homeostasis in late stage of DN.

In diabetes, vascular injury is induced by a variety of circulating toxins, namely, high glucose [15], oxidative stress [6], cytokines [7], altered shear stress [4, 5], thrombin [16], and angiotensin II (Ang II) [8]. Altered vascular homeostasis observed in late stage of DN is characterized by both defective angiogenic factors, namely, VEGF, VEGFR1, endothelial progenitor cells, and angiopoietin 1, as well as abnormally elevated antiangiogenic factors, namely, VEGFR2 and angiopoietin 2 [17]. Such changes would incriminate in mixed pictures of an insufficient, physiologic vasculogenesis, and of a pathologic progression of vascular disease resulting in a progressive reduction in vascular perfusion to the kidney as follows (Figure 3).

(I) With respect to VEGF, we have studied VEGF value in the serum, which partly reflects a component of circulating endothelial cell-receptor-bound VEGF detached from the diseased vascular wall into the circulation. Our study demonstrated a wide range of VEGF values with the mean value of VEGF not significantly different from the control. This observation of VEGF in diabetic patient is quite contrast to the reduced VEGF value observed in the nondiabetic chronic kidney disease patient [18]. Furthermore, several reports on changes in VEGF gene expression in kidney tissue are interesting. Bortolosa et al. [19] demonstrated a significant inverse correlation between total intraglomerular VEGF in RNA level and albumin excretion rate. Both the degree of mesangium and mesangial matrix expansion were inversely related to VEGF 165 and directly related to VEGF 121 in RNA levels. In addition, a strong inverse correlation between VEGF 165 and VEGF 121 isoforms was also found. Hohenstein et al. [9] demonstrated that VEGF expression was increased in all diabetic glomeruli by many different cell types. In contrast, VEGF receptor activation or receptor-bound VEGF was increased predominantly in the endothelium of only mildly injured glomeruli but not severe diabetic lesions. Cooper et al. [20] suggested that although VEGF-A may be elevated in the initial phase of diabetic nephropathy, it may not be maintained as more chronic fibrotic changes occur in the kidney. Indeed, in many animal models of chronic kidney disease, VEGF-A levels are reduced, correlating with the progression of renal damage [21, 22]. The different expressions of VEGF gene in different stages of DN is, therefore, dependent upon the dynamic balance between the degree of vascular injury and the stage of disease resulting in the detachment of receptor-bound VEGF from the diseased vascular wall into the circulation, and the ability to maintain the vascular repair by releasing the VEGF from the remaining sources such as podocyte, tubular cell, pericyte, and so forth. 

(II) With respect to defective VEGFR1 documented in this study in late stage of DN, the activation of VEGF through the classical VEGF → VEGFR 1 pathway would be defective, impairs physiologic Akt phosphorylation, uncoupling of eNOS, and thus impairs NO production. Previous study confirmed this view in vitro when VEGFR 1 was knocked down in endothelial cells using retroviral small interfering RNA for VEGFR 1, but not VEGFR 2; the cGMP was lower in VEGFR 1 knocked down endothelial cells, suggesting a decreased NO bioavailability [23]. 

(III) With respect to the defective endothelial progenitor cell and angiopoietin 1, diabetic patients have been reported to have a reduced number of circulating endothelial progenitor cell as well as its function, with the extent of reduction directly proportional to plasma hemoglobin A1C level [24, 25]. A defective endothelial progenitor cell in conjunction with an impaired NO production would impair the physiologic stimulation of endothelial cell proliferation and thus inadequately replace for the endothelial cell loss during vascular injury. The defective angiopoietin 1 would also impair the maturation and the maintenance of the integrity of the mature vessels. Previous study demonstrated a reduced angiopoietin 1 at 8 weeks following streptozotocin injection in adult rats  [26]. In addition, the increased expression of antiangiogenic factor angiopoietin 2, triggered by high sugar and angiotensin II, would further suppress the angiopoietin 1, then destabilize glomerular endothelium, and induce endothelial apoptosis [15, 27, 28]. In human and animal diabetic nephropathy, increased angiopoietin 2 expression was also observed by several investigators, namely, Rizkalla et al. [26], Yamamoto et al. [29], and Woolf et al. [30]. Collectively, these would incriminate the impaired physiologic vascular repair and induce insufficient vasculogenesis. This finding correlates well with the altered renal hemodynamic study, which reveals a progressive reduction in peritubular capillary flow in late stage of DN [2], a persistency of chronic ischemic state of the tubulointerstitial structure, and a reduction in peritubular capillary densities shown by platelet-endothelial cell adhesion molecule-1/CD 31 staining [31]. In contrast to the defective activation through the angiogenic VEGFR1 pathway in late stage of DN above, the activation through the antiangiogenic VEGFR2 pathway is excessively exaggerated as follows.

(IV) With respect to the abnormally elevated antiangiogenic VEGFR2 observed in late stage of DN, other studies showed conflicting results. Cooper et al. [20] demonstrated that VEGFR2 was elevated in short-term diabetes, whereas VEGFR2 was unaltered in long-term diabetic animals. However, Sasso et al. [32] demonstrated a reduction of both VEGFR1 and VEGFR2 expressions as well as a reduction of VEGFR2 phosphorylation in the myocardium of diabetic patients compared with nondiabetic patients. To explain such discrepancy, the observation in the cardiac myocardium may (i) not reflect the actual change of vascular homeostasis occurring in the diseased coronary vessel, but simply reflect the change in the underlying diseased myocardium under the environment associated with a defective collateral microcirculation in the heart and (ii) reflect the advanced ischemic disease in myocardium which is similar to the change observed in advanced stage of chronic ischemic renal disease. In this regard, increased microvascular rarefaction in the advanced disease of myocardium is similar to the peritubular capillary rarefaction observed in the advanced stage of tubulointerstitial fibrosis in chronic kidney disease [31, 33]. Under such advanced stage, there has been a continuous process of vascular injury associated with a progressive loss of endothelial cell-receptor-bound VEGF attached to the diseased vessel into the systemic circulation.

In accordance with the increased VEGFR2 observed in late stage of DN, it would pathologically activate Akt phosphorylation through the NO-independent pathway to induce endothelial cell proliferation and endothelial cell dysfunction. Previous study demonstrated that this excessive Akt activation under an impaired NO production would instigate the angiogenic response to VEGF, negatively regulat endothelial cell lifespan and inhibiting endothelial tubular formation via a p53/p21-dependent pathway [11]. In the presence of the defective angiopoietin 1 inducing an immature endothelial cell proliferation, in conjunction with the elevated angiopoietin 2 destabilizing the endothelial cell and enhancing endothelial apoptosis, these would collectively induce an abnormally immature endothelial cell proliferation. This cell would be consistent with the endothelial-myofibroblast transition cell as proposed by Li and Bertram [34]. To explain the vascular smooth muscle cell (VSMC) proliferation, the upregulations of vasoconstrictor (angiotensin II), procoagulant proteins, cytokines, and adhesion molecules in conjunction with the intraglomerular hypertension secondary to glomerular endothelial cell dysfunction have been documented in a variety of chronic kidney diseases and DN [5, 35]. Angiotensin II activates NADPH oxidase, induces reactive oxygen species and NK-*π*B activation, then activates ERK-p38, JAK-STAT, and therefore induces VSMC proliferation. Moreover, the increase in transmural pressure associated with intraglomerular hypertension would, in accordance with La Place's law, translate into an increase in biaxial tensile stress which then transmit into the wall of arteriole and enhance further the VSMC proliferation in the vascular wall. The VSMC proliferation would induce a thickening of vascular wall, a narrowing of vascular lumen, and eventually a progressive reduction in peritubular capillary flow. Collectively, these would incriminate in the pathologic development of neoangiogenesis, a progressive macro- microvascular disease. In this regard, Osterby and Nyberg [36] described abnormal blood vessels in glomeruli of patients with long-term type 1 diabetic, as well as type 2 diabetic patients [37]. Min and Yamanaka [38] demonstrated through analyses of computer-generated three-dimensional images in 94 diabetic nephropathic patients and found abnormal vessels anastomosed to the lobular structure of the intraglomerular capillary network, mainly to afferent branches, while the distal end of the vessels connected to the peritubular capillary. In these vessels, native endothelial cell function was likely impaired, with the endothelial cells initially swollen and endothelial thickness gradually decreasing as diabetes progressed [39, 40].

(V) With respect to altered vascular homeostasis and angiogenesis in diabetic retinopathy, expressions of VEGFA, VEGFR1, and VEGFR2 were all increased in the retina of diabetic or insulin-resistant rats [41]. Such change mimics the altered vascular homeostasis observed in early or mildly injured glomeruli. It is likely that in this circumstance of elevated VEGFR2, the VEGFR1 activity would be unable to suppress the VEGFR2. Therefore, the enhanced VEGF2 activity would pathologically stimulate Akt phosphorylation through the NO-independent pathway (uncoupling eNOS), and induce pathologic proliferation of abnormal, immature endothelial cell. Mice with homozygous mutations that inactivate either receptor (VEGFR1 or VEGFR2) die in utero with a similar phenotype as mice with VEGF deletion, indicating that both receptors are obligatory for the function of VEGF [42, 43]. Although the vascular phenotype between these VEGFR mutant mice are overlapping to some degree, they do differ in terms of showing that VEGFR2, may play a greater role in (pathologic) vascular organization [44]. Scott McLeod demonstrated a strong expression of KDR/Flk1 by proliferating endothelial cells in reforming retinal vessels and intravitreal neovascularization after hyperoxic insult in dog. Anti-KDR antibody delivered by slow-release pellets had no effect on normal vasculogenesis, but it inhibited the formation of intravitreal neovascularization and retinal vessel development in oxygen-induced retinopathy [45].

## 5. Vascular Homeostasis in Early Stage of DN and Therapeutic Implication

In contrast to the abnormal vascular homeostasis and angiogenesis observed in late stage of DN, our study demonstrated the values of vascular homeostasis observed in early stage of DN (normoalbuminuria) not significantly different from the control population [46]. This implies that this normal vascular homeostasis would reflect an adequate function of vascular repair. Theoretically, it would adequately induce the Akt phosphorylation, coupling of eNOS, and thus enhance NO production. Enhanced NO production would allow a normal response to vasodilators treatment resulting in vasodilating the underlying renal vasculature and restoring renal perfusion. Indeed, increment in peritubular capillary flow is documented following vasodilators treatment in early stage of DN during the stage of normoalbuminuria [47, 48]. Increased peritubular capillary flow following the relaxation of efferent arteriole not only inhibits the mechanism of tubulointerstitial fibrosis, but also induces renal regeneration. This is reflected by the regression of FE Mg following treatment with vasodilators, since FE Mg value correlates with the magnitude of tubulointerstitial fibrosis. Simultaneously, it also increases glomerular filtration rate following the relaxation of afferent arteriole. 

In conclusion, the preceding information renders support that implementation of treatment at the early stage of DN under the environment favorable for renal angiogenesis and regeneration would effectively restore renal function and prevent the end-stage renal disease.

## Figures and Tables

**Table 1:** Clinical findings in early stage of type 2 DN (normoalbuminuria).

	Pretreatment	Normal	*P* value
Renal function			
S Cr mg/dL	0.9 ± 0.5	<1	<.05
MA/Cr *μ*g/mg	13 ± 5	<30	NS
CCr ml/min/1.73 m^2^	84 ± 24	<120	<.01
FE Mg	3.5 ± 1.3	<2.2	<.05

Hemodynamics			
Futrakul (2007)			
PTCF mL/min/1.73 m^2^	292 ± 41	485 ± 39	<.01
GFR mL/min/1.73 m^2^	88 ± 28	119 ± 15	<.001

**Table 2:** A predominant role of VEGFR2 correlates with PTCF reduction in late stage of DN.

	Vascular homeostasis				Renal hemodynamics		
	Early DN	*P* value	Normal		Early DN	*P* value	Normal
VEGF R1 ng/mL	60±12	NS	49 ± 5	PTCF mL/min/1.73m^2^	379 ± 70	<.05	483 ± 43
VEGF R2 ng/mL	5715 ± 1400	NS	6126 ± 1066				

	Late DN	*P* value	Normal		Late DN	*P* value	Normal
VEGF R1 ng/mL	33 ± 17	<.01	55 ± 11	PTCF mL/min/1.73m^2^	277 ± 80	<.001	483 ± 43
VEGF R2 ng/mL	10414 ± 2198	<.01	7696 ± 1892				
